# Integrating Immersive Virtual Reality Meditation into Palliative Oncology: A Randomized Trial Protocol for Evaluating Pain Relief and Quality of Life

**DOI:** 10.3390/healthcare14020266

**Published:** 2026-01-21

**Authors:** Emily Santos Montarroyos, Sara Lima, Raimundo Barreto, Rosana Moysés, Letícia Zumpano Cardenas

**Affiliations:** 1 A.C. Camargo Cancer Center, Graduate Program in Oncology, 211 Professor Antônio Prudente Street, Liberdade, São Paulo 01509-001, SP, Brazil; emilymontarroyos@hotmail.com; 2 Pain Therapy and Palliative Care Service, FCECON, 215 Francisco Orellana Street, Planalto, Manaus 69040-010, AM, Brazil; rosana.pimentelcorreia@gmail.com; 3 Innovation in Health and Well-Being Research Unit (iHealth4Well-Being), Tâmega e Sousa School of Nursing, Northern Polytechnic Institute Health (IPSN-CESPU), Direita Rua, 5, 4560-485 Penafiel, Portugal; 4 Institute of Computing, Federal University of Amazonas, General Rodrigo Octávio Avenue, 6200, Northern Sector, University Campus, Coroado, Manaus 69080-900, AM, Brazil; rbarreto@icomp.ufam.edu.br; 5 Department of Dentistry, Unifeso–Serra dos Órgãos University Center, Alberto Tôrres Avenue, 111-Alto, Teresópolis 25964-004, RJ, Brazil; 6 Department of Physiotherapy, A.C. Camargo Cancer Center, 753 Tamandare Street, Liberdade, São Paulo 01525-001, SP, Brazil; leticia.cardenas@accamargo.org.br; 7 Intensive Care Department, A.C. Camargo Cancer Center, 211 Professor Antônio Prudente Street, Liberdade, São Paulo 01509-001, SP, Brazil

**Keywords:** oncological pain, cancer pain, quality of life, meditation, virtual reality, palliative care

## Abstract

**Highlights:**

**What are the main findings?**

- Immersive virtual reality can be used as a meditation tool for cancer patients in palliative care.
- The randomized clinical trial protocol evaluates its effectiveness in pain management and quality of life.

**What are the implications of the main findings?**

- Virtual reality meditation may provide a complementary strategy to improve well-being in integrative oncology.
- The study could generate scientific evidence to support better healthcare decisions in cancer pain management.

**Abstract:**

**Background/Objectives**: Cancer is a disabling, challenging, and growing global disease. Although early diagnosis and adequate treatment of cancer are developing rapidly, a large part of the population remains without access to specialized services and routinely progresses to uncontrolled pain, poorer quality of life, and suffering. Complementary therapies for pain management and the well-being of patients under palliative care are fundamental tools of integrative oncological medicine. This first version of the protocol was created in August 2023 to structure the aim of this study to investigate the effectiveness of the experimental protocol which uses immersive virtual reality as a meditation tool in patients followed at the Pain Therapy and Palliative Care Service of the CECON Foundation. **Methods**: This randomized clinical trial, conducted at the Pain Therapy and Palliative Care Service (STDCP) of the FCECON, explores the use of immersive virtual reality to promote regular meditation practice among cancer patients as an effective means of managing pain and improving quality of life. **Discussion**: The present study has the potential to evaluate the effectiveness of immersive virtual reality as a meditation tool for patients undergoing palliative care, in addition to contributing scientific evidence that supports better decisions in healthcare for the management of cancer pain. Trial registration: Brazilian Registry of Clinical Trials (ReBEC) and ClinicalTrials.gov/NCT06328751/Universal Trial Number (UTN) U1111-1304-3752.

## 1. Introduction

The increase in cancer incidence and mortality remains a global challenge with projections indicating a substantial increase in incidence over the coming decades that will impact health systems already facing ongoing structural limitations [1].

Brazil recorded 627,193 new cancer diagnoses in 2022 and projections indicate that by 2045 the increase will reach approximately 69%, in comparison to 2022 [1], placing cancer among the leading causes of morbidity and mortality in the country, presenting one of the greatest challenges to the public health system [2]. Given these regional and national differences, it is important to define a strategy to allow for equity in access to cancer healthcare [3] since unequal screening coverage and the limited number of specialized cancer centers lead to late diagnoses, reduced survival rates, increased costs, morbidity, and an impact on the quality of life of these patients [2].

Despite significant advances in oncological therapies, pain remains one of the most prevalent and distressing symptoms experienced by patients with cancer [4]. For many patients, pain is the first symptom of the disease, and most experience moderate to intense pain throughout their illness, making it one of the most feared and uncomfortable symptoms reported by cancer patients [5]. A clearer understanding of the mechanisms driving cancer-related pain, along with the integration of effective pain therapies into routine oncological care, is essential to improving both quality of life and survival among patients with cancer [6].

Cancer pain is diverse and multifactorial and results from a set of interactions related to tumor mechanisms such as inflammatory and neuropathic mechanisms and may also be associated with the effects of treatment [7]. Although there is strong evidence that the experience of pain can negatively affect clinical outcomes at all stages of the disease, cancer patients do not always receive adequate pain treatment [6,7].

The World Health Organization’s Analgesic Ladder continues to play a key role in pain treatment and guides pain management according to its intensity [4,8]. However, these guidelines do not address the heterogeneity of mechanisms in patients with different types of cancer, disease stages, and treatment plans. Thus, pain remains a frequent complaint for a significant portion of patients who experience incomplete relief or side effects that limit the dose of medications available in cancer centers and require complementary approaches that address both the physical and psychosocial dimensions of suffering [7,8,9].

Palliative care is an approach to improving the quality of life of patients with life-threatening illnesses that cause enormous family distress [9]. Instead of focusing exclusively on treatments that modify the disease, palliative care prioritizes symptom control, psychosocial support, and the preservation of dignity, meeting the needs of patients and their families throughout the course of the disease [8,9].

Mindfulness is a term that refers to the practice of being intentionally and non-judgmentally attentive in the present moment. Mindfulness meditation is the central component of mindfulness-based interventions that involves the practice of meditation and mindfulness techniques, and it is a skill that can be trained and has several modalities for its development, which may involve breathing techniques, music/sounds, and guided imagery, which encourage a deep state of realignment, attention, and balance of the body and mind [9,10,11].

For individuals experiencing chronic pain, the regular practice of mindfulness has been shown to have consistent and far-reaching positive effects on various aspects of daily life and health [9]. This intervention promotes a reduction in pain perception and contributes to significant improvements in overall functional activity, mood balance, and physical capacity, including walking and performing work activities [10]. Additionally, it positively impacts the quality of interpersonal relationships, sleep patterns, and subjective well-being and life satisfaction [10,11,12].

In individuals living with chronic pain, the practice of mindfulness is associated with reduced pain-related suffering and improvements in functional performance, emotional regulation, sleep quality, and well-being, as there are changes in attention processing and modulation of the neural circuits involved in stress and pain perception [11,12].

Recent studies have examined novel methodologies for mindfulness practices in the context of cancer pain management, with interventions facilitated by digital technologies emerging in a promising trend [13]. By combining guided meditation with immersive audiovisual environments, VR-based interventions may enhance attentional engagement and facilitate transient analgesic effects through mechanisms related to distraction and emotional regulation. Preliminary studies have reported benefits such as increased engagement, promotion of relaxation, and transient pain relief; however, considerable methodological challenges and difficulties related to the usability of these resources persist [14].

Despite the recent surge in research investigating the use of VR-mediated meditation, particularly in cancer patients receiving palliative care, there is an ongoing need for more robust investigations to assess its effectiveness, associated clinical outcomes, and the duration of its effects. Studies suggests that immersive virtual reality may act through multiple neurophysiological mechanisms, particularly those related to conditioning, attention modulation, and therapeutic exposure processes, contributing to the management of cancer pain [15,16].

Thus, the general objective of this study is to investigate the effectiveness of an experimental protocol that uses immersive virtual reality as a meditation tool in patients followed at the Pain Therapy and Palliative Care Service of the CECON Foundation.

## 2. Materials and Methods

### 2.1. Trial Design

**Figure 1:**
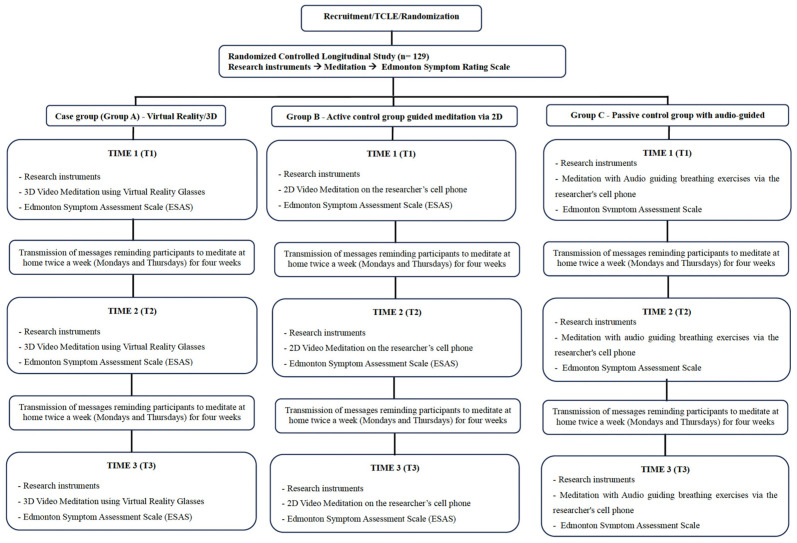
Flowchart of the experimental design.

The protocol uses an experimental, longitudinal, randomized controlled trial (RCT) approach, conducted at three time points. The experimental design is shown in the flowchart in Figure 1, and the temporal implementation of the experimental protocol is demonstrated in Table 1.

### 2.2. Study Setting

This project was approved on 5 January 2024 by the FCECON Research Ethics Committee under the number CAAE: 76043823.0.0000.0004 and subsequently registered on the following Research Registration Platforms: Brazilian Registry of Clinical Trials (ReBEC) under number 15,398 and ClinicalTrials.gov/NCT06328751/Universal Trial Number (UTN) number U1111-1304 3752.

The research is conducted in the medical office, used by the medical team to care for patients monitored by the Pain Therapy and Palliative Care Service (STDCP) of the Amazonas State Oncology Center Foundation (FCECON). The STDCP has a multidisciplinary team of anesthesiologists specializing in pain therapy, palliative medicine, and acupuncture, nurses, psychologists, physical therapists, chaplains, and nursing technicians. All research processes are carried out in rooms used by the STDCP team and conducted by the team responsible for the protocol. This team includes doctors and medical students trained to explain all phases of the study, its risks, and its benefits; ensure confidentiality before, during, and after the research; and provide compensatory assistance to those who suffer harm from participating in the study.

### 2.3. Recruitment and Participants

The protocol includes patients between the ages of 18 and 75 who are assisted at the STDCP, with a diagnosis of cancer pain; who can understand Portuguese, have normal vision and hearing, can move their heads, and have sufficient motor control to perform bodily movements; who have a mobile phone with a compatible system; and who agree to participate in the study and sign the informed consent form (ICF).

Indigenous patients were excluded from study due to cultural peculiarities, language, and special legislation. The other exclusion criteria are patients with a history of serious mental illness (DSM-5, Diagnostic and Statistical Manual of Mental Disorders—schizophrenia, schizotypal disorders, delusional disorders, borderline personality disorder, manic phase in bipolar disorder, and dementia); neurological deficits that compromise the degree of cooperation (Glasgow Coma Scale ≥ 14); impaired comprehension and communication skills based on the researcher’s assessment; reports of discomfort when using immersive virtual reality; disease progression that limits regular outpatient mobility; and patients with brain tumors, brain metastases, metastatic lung neoplasm, or a history of seizures.

In order to participate in the study, patients are required to provide informed consent. The consent document includes a comprehensive explanation of the study’s objectives, its potential risks and benefits, and the various stages of the research process. The subjects are then requested to provide their consent and written informed consent is then obtained from all participants.

The protocol stipulates that harm will be defined if any adverse effect occurs after randomization of study participants. The harm will be evaluated systematically at all time points of the study (T1, T2, T3) through the use of structured questionnaires and symptom scales, as well as non-systematically through spontaneous reports from participants. All events will be documented, and serious adverse events will be reported to the ethics committee, as required by Brazilian ethical standards. The ICH Good Clinical Practice (ICH-GCP) definitions will be used to categorize all adverse events.

The protocol does not utilize an Independent Data Monitoring Committee, as this randomized controlled protocol uses non-invasive behavioral interventions (guided meditation via virtual reality, video, or audio) and does not include any procedures or treatments associated with significant safety concerns or potential for adverse events. Therefore, it is not expected to cause serious adverse events.

The responsibility for conducting safety monitoring lies with the research team, in accordance with the Good Clinical Practice guidelines. The nature of the study does not permit interim analyses; consequently, there is no definition of early termination guidelines or statistical stopping rules. Consequently, preliminary data analyses will not be disseminated to members of the research team or to external parties.

In accordance with the principles of ethics and regulatory compliance, the dissemination of anonymised individual participant data is strictly prohibited, and the publication of study results will be limited to a purely aggregated form. It is important to note that the study may be terminated prematurely in the event of any unforeseen safety concerns or at the discretion of the Research Ethics Committee. In such cases, the ultimate decision shall be made by the principal investigator, following consultation with the ethics committee.

### 2.4. Study Assessment Instruments

For this protocol, we use the following instruments:

(1) Sociodemographic and Clinical Questionnaire: This questionnaire was developed for this study to assess the following sociodemographic variables: age, education, religion, origin, race, marital status, occupation, and participants’ monthly income. It also assesses the following clinical variables: patient age, diagnosis, staging, and types of treatment, which are obtained from patients’ medical records.

(2) McGill Quality of Life Questionnaire [17]: This 16-item questionnaire is divided into domains such as physical well-being, psychological well-being, existential well-being, support, and physical symptoms. It presents a single-item scale that measures overall quality of life, which is not included in the total score but rather is used for comparison. There is also an open-ended question in which patients can list the things that have had the greatest effect on their quality of life. The scale has 11 points, ranging from 0 to 10. A higher final score indicates a better quality of life.

(3) Brief Pain Inventory (Reduced Version) [18]: This questionnaire uses a body diagram on which patients can mark the location of their pain. There are also eight questions about pain intensity on a scale from 0 to 10. A higher inventory score indicates worse pain intensity.

(4) Hospital Anxiety and Depression Scale (HADS) [19]: This is a 14-item instrument with two subscales, one for anxiety and one for depression, each with 7 items. Each item’s score can range from 0 to 3, with a maximum score of 21 points for each subscale. Patients without anxiety or depression are considered to have a score from 0 to 8, while those with a score of 9 or higher are considered to have anxiety or depression

(5) Edmonton Symptom Assessment Scale (ESAS) [20]: This is a self-report questionnaire designed to measure both objective and subjective symptoms. In this scale, patients or their caregivers/family members assign a score from 0 to 10 to each symptom, with 0 representing the absence and 10 representing the highest intensity of the symptom.

(6) National Comprehensive Cancer Network^®^ (NCCN) Distress Thermometer [21]: This is administered in the form of a questionnaire during the three designated moments. This instrument, which has been translated and validated by the NCCN, is utilized to assess the psychological well-being of individuals diagnosed with cancer.

In each interview (T1, T2, and T3), all questionnaires are administered before the meditation session (Before Meditation), and only the ESAS is administered immediately after the meditation session (After Meditation) to capture acute changes in symptoms. All assessment instruments are based on questionnaires and assessment scales already validated in the literature and are listed in Table 2.

### 2.5. Interventions

The study is conducted at three moments (T1, T2, and T3) with all study groups (case group—Group A; active control group—Group B; passive control group—Group C). In T1 of the study, all groups respond to the baseline assessment approach of the study, which consists of a sociodemographic and clinical questionnaire for the three groups. The data is collected at the FCECON office, immediately after these patients have completed their medical appointment at the Pain Therapy and Palliative Care Service at FCECON.

Participants are randomized into three groups: a case group with guided meditation using virtual reality/3D (three-dimensional) video (Group A), an active control group with guided meditation using 2D (two-dimensional) video (Group B), and a passive control group with guided meditation using audio (Group C). All participants are interviewed at three moments: time 1 (T1)—the first day of intervention and collection of instruments; time 2 (T2)—four weeks after T1; time 3 (T3)—eight weeks after T1.

In all groups and at all moments, T1, T2, and T3, patients respond to the sociodemographic and clinical questionnaire and other instruments measuring quality of life, psychological morbidity, pain, and symptoms. After completing the meditation, patients respond to the Edmonton Symptom Assessment Scale.

The randomization sequence and the type of meditation used in each group at the FCECON office are accessible to the research team, but participants are not aware of the other research groups or the type of meditation is used in each group. Data collection takes place separately on different days and at different times, without one participant having access to another’s meditation.

Subsequently to the in-person intervention at the FCECON office, participants from all three groups receive standardized instructions for conducting home meditation. The practice should be conducted using a video previously provided by the research team, accessed on the participants’ own cell phones, without the need for an internet connection, in offline mode.

The content of the home meditation consists of the same 2D video with guided meditation audio for Groups A and B and Group C receives only audio for guided meditation. In order to promote adherence to the protocol, all participants receive standardized reminders on their mobile devices requesting that they perform the meditation on Mondays and Thursdays between 9 am and 10 am during the eight weeks of the experimental protocol.

#### 2.5.1. Case Group (Group A)

This group performs meditation with a virtual reality (VR)/3D (three-dimensional) video: patients watch a 10 min immersive virtual reality video of mindfulness meditation with images and sound in a comfortable chair at the FCECON office, using Samsung^®^ Gear VR virtual reality glasses and a Samsung^®^ smartwatch.

The meditation for Group A consists of watching an immersive 3D virtual reality (VR) video in 360°, entitled “Secret Garden,” where participants can immerse themselves in a beautiful and relaxing Japanese garden, with all the natural elements, such as the flow and sound of running water. This experience was made available by the Italian company BECOME SRL, also known as BECOME. Augmented Life, available at “www.discoverbecome.com”. BECOME SRL is a startup specializing in creating customized virtual reality content and environments for researchers and neuroscientists. The video used in our study was developed especially for this research, with audio in Brazilian Portuguese.

#### 2.5.2. Active Control Group (Group B)

This group performs meditation with 2D video (two-dimensional video): patients watch a simple 10 min video presented via cell phone with image and sound, featuring mindfulness meditation in a comfortable chair in the FCECON office, using a Samsung^®^ smartwatch.

The meditation in Group B consists of watching a 2D video in non-immersive mode on an Android cell phone. Participants in this group then have the opportunity to observe a beautiful and relaxing lake surrounded by a forest to the sound of meditation guided by the psychiatrist, psychotherapist, and mindfulness teacher Dr. Marcelo Tombka. This video is part of the video collection used in Dr. Marcelo Tombka’s research and was made available for use in our study.

#### 2.5.3. Passive Control Group (Group C)

This group performs meditation with an audio-guided meditation: Audio for breathing exercises Time 1. Patients perform breathing exercises based on instructions from the healthcare professional for 4 min in a comfortable chair at the FCECON office, using a Samsung^®^ smartwatch.

The meditation in Group C consists of listening to an audio recording in non-immersive mode on an Android cell phone, where participants have the opportunity to perform breathing exercises to the sound of meditation also guided by the psychiatrist, psychotherapist, and mindfulness teacher Dr. Marcelo Tombka. This audio recording is part of Dr. Marcelo Tombka’s research audio collection, which was made available for use in our study.

#### 2.5.4. Randomization and Blinding

The randomization is performed by the principal investigator using a specific commercial mobile application called Randomizer^®^ for Clinical Trial Lite, which is a secure, validated application that works with encrypted network traffic using TLS (Transport Layer Security), a strong encryption for random distribution among the research groups.

The study is a comparative study; the participants in the case group, active control group, and passive control group are recruited in the same way, receive the same information about meditation, and answer the same questionnaires before and after meditation. However, given the nature of behavioral interventions (virtual reality and 2D video or audio), both participants and research assistants conducting the interventions cannot be blinded to group allocation. The questionnaires used in the study are administered and collected by researchers who are aware of the intervention modality and are responsible for setting up the equipment, training participants, and providing technical support.

To minimize bias, the statistician responsible for the analyses will remain blinded to group allocation, and group labels will be coded during data analysis. We recognize the lack of blinding of the evaluator as a limitation, which will be considered in the interpretation of the results.

### 2.6. Outcome Measures

The primary outcome of the protocol is the change in pain intensity, measured by the Brief Pain Inventory—Short Form (BPI), from the beginning of the study (T1) to the eight-week follow-up (T3). The item “worst pain in the last 24 h” (numerical scale from 0 to 10) will be used as the indicator of pain intensity, as it has been validated and demonstrated to be clinically important in oncology and palliative care populations.

Secondary outcomes will encompass metrics designed to assess the psychological, functional, and symptomatic domains. Psychological morbidity will be assessed using the Hospital Anxiety and Depression Scale (HADS), including total and subscale scores. Quality of life will be evaluated using the McGill Quality of Life Questionnaire.

In addition to longitudinal assessments at T1 (baseline), T2 (four weeks), and T3 (eight weeks), the immediate pre- and post-intervention effects will be explored using the Edmonton Symptom Assessment Scale (ESAS). The ESAS will be administered prior to and following each in-person guided meditation session to assess acute changes in symptoms. Pain intensity, severity, and interference will be measured using the respective subscales of the BPI. The extent to which participants adhere to the intervention will be determined by calculating the percentage of prescribed meditation sessions completed during the follow-up period.

The subsequent Table 3 offers a synopsis of all outcome measures, variables, analysis metrics, aggregation methods, and assessment time points.

## 3. Statistical Analysis

### 3.1. The Sample Size

The sample size was determined based on the estimated number of approximately 140 patients monitored monthly at the FCECON Pain Therapy and Palliative Care Service. In the absence of prior evidence to facilitate reliable estimates of effect size, variance, or intra-participant correlation for comparisons between immersive virtual reality, 2D video, and guided meditation in adults with cancer-related pain receiving palliative care, a formal power-based calculation could not be conducted. Consequently, the sample size was determined using a feasibility-based approach. In the context of palliative oncology, where recruitment is inherently limited and attrition rates are high, using the average monthly number of eligible patients to determine sample size is an appropriate strategy.

The sample calculation was performed using G*Power 3.1 software. For the calculation, an effect size of 71% was considered for three groups. For the remaining parameters, a significance level of 5% and a sample power of 96% were adopted. These parameters were also defined for the three groups, under the assumption that there would be three measurement moments and that the analyses would be performed in SPSS (version 29.0).

The sample size was determined to be 39 participants per group. Consequently, the total number of participants would be 117, categorized into three groups of 39 participants each. Furthermore, considering the possibility of loss to follow-up, 12% was added to the final value of 39 participants, resulting in a final sample of 129 participants.

The final sample size was divided into three groups: an experimental group (Group A), a passive control group (Group B), and an active control group (Group C). The total number of participants in each group was 43.

### 3.2. Analysis

Descriptive statistics are applied to characterize patients at baseline. Continuous variables are reported as the mean and standard deviation, while categorical variables are expressed as frequency and percentage. To analyze the longitudinal data of the three groups, repeated-measures ANOVA is used to define the impact of each intervention on the three paired groups of participants. The response variable, which is mindfulness meditation through virtual reality, is analyzed over time, comparing the three groups. If we do not have a normal sample, we will perform the analysis of Generalized Estimating Equations (GEEs), a semi-parametric method to test the differences between groups and the interaction of variables and groups over time.

## 4. Discussion

The present study proposes a differentiated approach, utilizing immersive virtual reality exclusively in the office as a tool for introducing and training in mindfulness, followed by home practice using 2D video. This approach aims to explore whether immersive virtual reality can aid in treatment adherence, reduce adverse events, and extend therapeutic effects beyond immediate analgesia. It is hoped that this will contribute to the debate on the existence of evidence supporting the incorporation of virtual reality as an educational and adjuvant resource in oncological palliative care [22].

The protocol proposed here stipulates that cancer patients in palliative care are responsible for maintaining the practice at home. According to the literature, the feasibility of meditation in virtual reality shows that adherence is higher when sessions are conducted in a supervised environment, while home practice presents a higher risk of abandonment [22,23]. In the context of palliative care in oncology, these factors assume even greater relevance, given that fatigue, clinical frailty, and adverse treatment effects can impede the continuity of the protocol, thereby constituting a methodological limitation [24].

Virtual reality (VR)-based non-pharmacological interventions (NPIs) for chronic pain produced reductions in pain intensity that, although they achieved minimal clinical significance in some outcomes, were not superior to equivalent multimedia applications on 2D screens [22]. These results partially contrast with the hypotheses of the present study, which proposes the use of immersive VR exclusively in an outpatient setting as a teaching and initial engagement tool for the practice of mindfulness meditation, which is subsequently performed at home through 2D videos.

Unlike the predominantly cognitive-distraction approach of comparative study, the approach delineated in this study seeks to evaluate the feasibility of virtual reality (VR) as a medium for facilitating learning and adherence to guided meditation, while exploring its potential therapeutic effects beyond immediate analgesia. By combining the immersive experience in the doctor’s office with regular mindfulness practice at home, the study examines whether this approach may be associated with more sustained outcomes in cancer-related pain, quality of life, and psychological morbidity (anxiety and depression).

While the existing literature demonstrates the benefits of VR in chronic pain contexts, with significant reductions in pain intensity, psychological distress, and functional disability, the present protocol seeks to expand this discussion of evidence to a scenario that has been little explored: cancer pain in palliative care [25,26].

Another aspect to consider is the emotional and psychological state of patients. Evidence indicates that emotional distress acts as a modulator of pain perception and response to non-pharmacological interventions [9]. Meditation can promote significant improvement in quality of life, as demonstrated even in populations with high psychological morbidity [10,23]. However, for these benefits to remain consolidated, continuous practice is essential, which makes adherence a critical variable.

Moreover, the extant literature acknowledges that in randomized trials, equivalence of duration between groups is not a methodological requirement. Consequently, these groups are capable of participating in sessions of varying durations, as the duration of interventions is no longer regarded as a limitation, in contrast to conventional mindfulness programs [26]. Studies have demonstrated that there are no substantial discrepancies between the short-term and long-term consequences of interventions that utilize brief meditation techniques to enhance well-being [27].

This scenario should be carefully considered in the final analysis of the results, since differences observed between modalities may reflect both the intrinsic effectiveness of each meditation format and the ability of each patient to maintain the proposed practice. Future research should explore strategies to optimize adherence, such as active remote support, hybrid sessions (in person and at home), or personalized adaptations to the patient’s clinical profile.

It is important to note that this protocol is vulnerable to certain limitations, particularly in cases involving patients receiving palliative care in the terminal phase of the disease. These limitations may include loss to follow-up due to death, the emergence of uncontrollable pain from disease progression, and the combination of pharmacological therapies such as chemotherapy, which can cause side effects and symptoms that are complex to manage.

## 5. Conclusions

The expected results from this protocol have the potential to provide robust evidence for the incorporation of mindfulness meditation as a complementary tool in pain management in palliative care, contributing to the strengthening of the integration of non-pharmacological interventions into established clinical practices. Furthermore, the meticulously outlined methodology in this study can serve as a reference for future research, fostering scientific reproducibility, adaptation to different healthcare contexts, and the continuous improvement of digital interventions applied to palliative care.

## Figures and Tables

**Table 1:** Temporal implementation of the experimental protocol.

Planning (0–1 Year)	Intervention (1–2.5 Years)
Questionnaire preparation Definition of criteria Patient selection Randomization and sample size Group allocation	Informed consent (TCLE) Initial questionnaire Assessments at 3 time points Meditation protocol Edmonton Symptom Scale

**Table 2:** Descriptive table of the experimental groups; time of the experimental protocol; and instruments applied.

Group	Time	Study Assessment Instruments
Case group (Group A) Virtual reality/3D (VR)	Time 1 (T1)—First Day of Intervention	
	Before Meditation	Sociodemographic and clinical questionnaire McGill Quality of Life Questionnaire [17] Brief Pain Inventory (Short Form) [18] Hospital Anxiety and Depression Scale [19] The Edmonton Symptom Assessment Scale (ESAS) [20] The National Comprehensive Cancer Network^®^ (NCCN) [21]
	After Meditation	The Edmonton Symptom Assessment Scale (ESAS) [20]
Active Control Group (Group B) Guided Meditation 2D Video	Time 2 (T2)—Four Weeks After T1	
	Before Meditation	McGill Quality of Life Questionnaire [17] Brief Pain Inventory (Short Form) [18] Hospital Anxiety and Depression Scale [19] The Edmonton Symptom Assessment Scale (ESAS) [20] The National Comprehensive Cancer Network^®^ (NCCN) [21]
	After Meditation	The Edmonton Symptom Assessment Scale (ESAS) [20]
Passive Control Group (Group C) Audio-guided meditation—Audio for breathing exercises	Time 3 (T3)—Eight Weeks After T1	
	Before Meditation	McGill Quality of Life Questionnaire [17] Brief Pain Inventory (Short Form) [18] Hospital Anxiety and Depression Scale [19] The Edmonton Symptom Assessment Scale (ESAS) [20] The National Comprehensive Cancer Network^®^ (NCCN) [21]
	After Meditation	The Edmonton Symptom Assessment Scale (ESAS) [20]

**Table 3:** Overview of outcome measures and analytical framework.

Outcome	Measurement Variable	Analysis Metric	Aggregation Method	Time Points
Primary outcome	BPI: “worst pain in the last 24 h” (0–10)	Change from baseline; final value	Mean (SD); mean difference	T1, T2, T3
Psychological morbidity	HADS total and subscales	Change from baseline	Mean (SD)	T1, T2, T3
Quality of life	McGill Quality of Life Questionnaire scores	Change from baseline	Mean (SD)	T1, T2, T3
Symptom burden	ESAS total score and individual items	Immediate post-session change and longitudinal change	Mean (SD)	T1, T2, T3
Pain-related symptoms	BPI severity and interference subscales	Change from baseline	Mean (SD)	T1, T2, T3
Adherence	Percentage of completed meditation sessions	Proportion	Mean proportion	Summary at T2 and T3

## Data Availability

The provision of the data presented in this study is prohibited on the basis of current ethical and regulatory restrictions in Brazil.

## Abbreviations

The following abbreviations are used in this manuscript:

FCECON	Amazonas State Oncology Control Centre Foundation
ReBEC	Brazilian Registry of Clinical Trials
STDCP	Pain Therapy and Palliative Care Service
VR	Virtual Reality
WHO	World Health Organization
RCT	Randomized Controlled Trial
UTN	Universal Trial Number
ICF	Informed Consent Form
ESAS	Edmonton Symptom Assessment Scale
TLS	Transport Layer Security
NPIs	Non-pharmacological Interventions
T1	Time 1
T2	Time 2
T3	Time 3
GEE	Generalized Estimating Equations
